# Benefits and harms of sodium‐glucose co‐transporter‐2 inhibitors (SGLT2‐I) and renin–angiotensin–aldosterone system inhibitors (RAAS‐I) versus SGLT2‐Is alone in patients with type 2 diabetes: A systematic review and meta‐analysis of randomized controlled trials

**DOI:** 10.1002/edm2.303

**Published:** 2021-10-12

**Authors:** Samuel Seidu, Setor K Kunutsor, Pinar Topsever, Kamlesh Khunti

**Affiliations:** ^1^ Diabetes Research Centre University of Leicester Leicester General Hospital Leicester UK; ^2^ National Institute for Health Research Bristol Biomedical Research Centre University Hospitals Bristol and Weston NHS Foundation Trust and the University of Bristol Bristol UK; ^3^ Translational Health Sciences Bristol Medical School Learning & Research Building (Level 1) University of Bristol Southmead Hospital Bristol UK; ^4^ Department of Family Medicine Acibadem Mehmet Ali Aydinlar University School of Medicine Kerem Aydinlar Campus Atasehir Turkey

**Keywords:** RAAS inhibitor, SGLT2 inhibitor, Type 2 diabetes

## Abstract

**Introduction:**

It is uncertain if the combination of sodium‐glucose co‐transporter 2 inhibitors (SGLT2‐Is) and renin‐angiotensin‐aldosterone system inhibitors (RAAS‐Is) provides better cardio‐renal clinical outcomes in people with type 2 diabetes mellitus (T2DM) compared with SGLT2‐Is alone. Using a systematic review and meta‐analysis of randomized controlled trials (RCTs), we evaluated the efficacy and safety with respect to cardio‐renal outcomes of the combination of SGLT2 and RAAS inhibitors vs SGLT2‐Is in patients with T2DM.

**Methods:**

Studies were identified from MEDLINE, Embase, the Cochrane Library and search of bibliographies to May 2021. The Cochrane risk of bias tool was used to assess the risk of bias of each study. Study‐specific risk ratios (RRs) with 95% confidence intervals (CIs) were pooled. Quality of the evidence was assessed using GRADE.

**Results:**

Nine articles comprising 8 RCT evaluations (*n* = 34,551 participants) that compared SGLT2‐Is with placebo in patients with T2DM against a background of standard care and reported subgroup results for those treated with or without RAAS‐Is at baseline were included. No RCT specifically investigated the combination of SGLT2 and RAAS inhibitors compared with SGLT2‐Is alone. The RRs (95% CIs) for composite cardiovascular outcome and composite CVD death/heart failure hospitalization comparing SGLT2‐Is vs placebo in patients on RAAS‐Is were 0.93 (0.85–1.01) and 0.88 (0.76–1.02), respectively. The corresponding estimates for patients not on RAAS‐Is were 0.78 (0.65–0.93) and 0.73 (0.65–0.82), respectively. There was no evidence of interactions between RAAS‐I status and the effects of SGLT2‐Is for both outcomes. Single study results showed that SGLT2‐Is vs placebo reduced the risk of composite kidney outcome and cardiovascular death in patients with RAAS inhibition. The effect of SGLT2 inhibition vs placebo on kidney parameters, genital infections, volume depletion, hyperkalaemia, hypokalaemia, hypoglycaemia and other adverse events was similar in patients with or without RAAS inhibition. The quality of the evidence ranged from very low to moderate.

**Conclusions:**

Aggregate published data suggest that the combination of SGLT2 and RAAS inhibitors in the treatment of patients with T2DM may be similar in efficacy and safety if not superior to SGLT2‐Is alone. Head‐to‐head comparisons of the two interventions are warranted to inform T2DM management. The use of SGLT2 inhibition as a first‐line therapy in T2DM or its early use in the prevention of renal deterioration and cardiovascular complications in addition to its glycaemic control deserves further study.

## Graphical abstract

The combination of SGLT2 and RAAS inhibitors in the treatment of patients with T2DM is similar to SGLT2‐I alone with respect to cardio‐renal outcomes. The safety profile of SGLT2‐Is alone is similar to the combination of SGLT2 and RAAS inhibitors in the treatment of patients with T2DM.

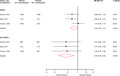

## 1 INTRODUCTION

Diabetes is a global public burden—it is a leading cause of morbidity, mortality and places substantial socioeconomic and financial pressures on individuals, health systems and global economies.^1^, ^2^ In 2015, diabetes (with type 2 diabetes being the most common type) was the sixth leading cause of disability.^3^ Chronic kidney disease (CKD), due to diabetic nephropathy, is a common complication in people with type 2 diabetes,^4^ with cardiovascular disease (CVD) being the leading cause of morbidity and mortality associated with type 2 diabetes. Currently, about 422 million people worldwide have diabetes^5^ and it has been projected that 592 and 642 million will have diabetes by 2035 and 2040, respectively.^6^, ^7^ People with type 2 diabetes need intensive management of glucose and risk factors such as lipids and blood pressure to reduce the risk of disease progression and complications.^8^ With the rising global tide of established risk factors such as obesity, physical inactivity and high energy diets, complications and deaths attributable to diabetes will proportionately increase if there is no concomitant improvement in its management.^6^ Lifestyle and metformin are the first‐line treatment of choice for patients with type 2 diabetes, unless contraindicated in specific situations such as those with advanced renal impairment.^1^ Because the kidneys are involved in the pharmacokinetic processing of many antidiabetic drugs^9^, ^10^, ^11^, ^12^ or their mechanisms of action,^13^ prescribing antidiabetic drugs in patients with type 2 diabetes and renal impairment can be very challenging. There are limited treatment options for glycaemic control in these patients.

Sodium‐glucose co‐transporter 2 inhibitors (SGLT2‐Is) (dapagliflozin, canagliflozin, empagliflozin and ertugliflozin) are the latest therapeutic agents for the treatment of type 2 diabetes. They increase excretion of glucose in the urine by inhibiting glucose reabsorption.^14^ Their use is associated with reductions in glycated haemoglobin (HbA1c) levels, systolic blood pressure (SBP), albuminuria and weight loss.^13^ There is substantial evidence that SGLT2‐Is reduce the risk of cardiovascular outcomes in those at high risk, the need for heart failure hospitalization and the progression of kidney impairment.^15^, ^16^, ^17^ SGLT2‐Is alone do not cause hypoglycaemia and exert beneficial effects without having significant adverse effects. Their main common side effect is genital mycotic infections.^18^ SLGT2‐Is are less effective for glucose control in patients with moderate‐to‐severe renal impairment (estimated glomerular filtration rate, GFR 30–60 ml/min/1.73 m^2^)^18^ and are not recommended in many guidelines for glycaemic control in people with estimated GFR less than 30 ml/min/1.73 m^2^. For several decades, renin‐angiotensin‐aldosterone system inhibitors (RAAS‐Is) (angiotensin‐converting enzyme inhibitors (ACE‐Is), angiotensin‐II type 1 receptor blockers (ARBs) and more recently direct renin inhibitors (DRIs)) have been employed to reduce the rate of progression of diabetes nephropathy in people with type 2 diabetes.^19^ Substantial evidence also suggests that RAAS‐Is reduce the risk of cardiovascular events.^19^, ^20^ SGLT2 and RAAS inhibitors each independently reduce the risk of cardiovascular and kidney complications associated with type 2 diabetes and they appear to have synergistic effects when used as combination therapy.^21^, ^22^ Hence, it will be clinically relevant to know the effectiveness of combining SGLT2 and RAAS inhibitors versus SGLT2‐Is alone. Though a number of landmark trials comparing SGLT2‐Is with placebo have reported outcomes among subgroups of patients with or without RAAS inhibition, no previous systematic review has synthesized the existing evidence. In this context, using a systematic review and meta‐analysis of randomized controlled trials (RCTs), we aimed to evaluate whether the combination of SGLT2 and RAAS inhibitors has a superior efficacy and safety profile than SGLT2‐Is alone in patients with type 2 diabetes.

## 2 METHODS

### 2.1 Data sources and search strategy

We registered this systematic review and meta‐analysis in the PROSPERO prospective register of systematic reviews (CRD42021251601). It was conducted using a predefined protocol and in accordance with PRISMA guidelines (Appendix 1). MEDLINE, Embase and the Cochrane Library electronic databases were searched from 2012 (being the year of approval of the first SGLT2 inhibitor (dapagliflozin) in the European Union) to 08 May 2021 with no restriction on language. The computer‐based searches combined terms related to the intervention (eg SGLT2 inhibitor, dapagliflozin, canagliflozin, empagliflozin and ertugliflozin), comparator (eg RAAS inhibitor, ACE‐I), ARB, DRI) and population (eg type 2 diabetes) in humans. A RCT design search filter was employed. Details on the search strategy are provided in Appendix 2. Titles and abstracts of all initially identified citations were initially screened by one author (SS) to assess their suitability for potential inclusion, followed by the acquisition of full texts for detailed evaluation. Full‐text evaluation was independently conducted by two authors (SS and SKK). The reference lists of key studies and review articles were manually scanned for additional studies.

### 2.2 Study selection and eligibility criteria

Randomized controlled, open or blinded trials that assessed the effects of the combination of SGLT2 and RAAS inhibitors compared with SGLT2 inhibitors in adults with type 2 diabetes and reported on renal or cardiovascular outcomes or adverse events were eligible. Randomized controlled trials that had also compared SGLT2‐I treatment with a placebo or standard care and reported outcomes according to whether patients were receiving RAAS‐Is or not at baseline were considered. We excluded the following: (i) studies that specifically enrolled only patients with known renal insufficiency or established renal parenchymal disease without diabetes mellitus and (ii) studies that recruited patients with a history of diabetic ketoacidosis, type 1 diabetes mellitus, history of hereditary glucose‐galactose malabsorption, primary renal glucosuria or renal disease that required treatment with immunosuppressive agents.

### 2.3 Data extraction

One author (SKK) initially extracted data from eligible studies using a predesigned data collection form and a second author (SS) independently checked the data with that in original articles. A consensus was reached in case of any inconsistency with involvement of a third (KK). Data were extracted on the following: first author, publication year, study year, specific study design, baseline population including duration of years with type 2 diabetes, proportion of men, geographical location, average age at baseline, numbers enrolled and randomized, allocation concealment, blinding, type of SGLT2‐I and dosage; duration of treatment or follow‐up; treatment comparisons; and nature of outcome events and their numbers. We extracted risk estimates when reported.

### 2.4 Outcomes

The primary outcomes were defined as (i) the 3‐point major adverse cardiovascular events (MACE), composite of death from cardiovascular causes, nonfatal myocardial infarction, or nonfatal stroke (composite cardiovascular outcome) and (ii) serum creatinine doubling, initiation of renal replacement therapy (RRT) or death from renal disease (composite renal outcome). Secondary outcomes were (i) cardiovascular death, (ii) heart failure (HF) hospitalization, (iii) composite outcome of cardiovascular death or HF hospitalization, (iv) decline in estimated GFR, (v) RRT, (vi) doubling of serum creatinine level, (vii) other renal and cardiovascular outcomes, (viii) glycaemic measures and haemodynamic and metabolic parameters, and (ix) adverse events.

### 2.5 Risk of bias

The risk of bias of each of the included trials was assessed using the Cochrane Collaboration's risk of bias tool.^23^ This tool evaluates seven possible sources of bias which are random sequence generation, allocation concealment, blinding of participants and personnel, blinding of outcome assessment, incomplete outcome data, selective reporting and other bias. For each individual component, studies were classified into low, unclear and high risk of bias.

### 2.6 Quality of evidence

We assessed the quality of the body of evidence on each outcome using the Grading of Recommendations Assessment, Development and Evaluation (GRADEpro) tool (https://gdt.gradepro.org), based on study limitations, inconsistency of effect, imprecision, indirectness and publication bias.^24^ We rated the quality as four levels: high, moderate, low and very low.

### 2.7 Statistical analysis

Summary measures of association were reported as risk ratios (RRs) with 95% confidence intervals (CIs). Risk ratios were pooled using a fixed effects model given the few studies available for pooling and the absence of substantial heterogeneity across studies. Standard chi‐square tests and the I^2^ statistic were used to quantify the extent of statistical heterogeneity across studies.^25^, ^26^ We employed random effects meta‐regression to assess for interactions between RAAS inhibition status and the effect of SGLT2‐Is.^27^ Only two meta‐analysis could be carried out due to limited data. Given the variety of measures reported for some outcomes and inconsistent reporting by some of the trials, a formal meta‐analysis could not be performed for some of the outcomes. A narrative synthesis was performed for studies that could not be pooled. The findings of such studies were summarized in tables that included the main characteristics of the study and the results in natural units as reported by the investigators. All tests were two‐tailed, and p‐values of 0.05 or less were considered significant. All analyses were conducted using Stata version MP 16 (Stata Corp).

## 3 RESULTS

### 3.1 Study identification and selection

**FIGURE 1:**
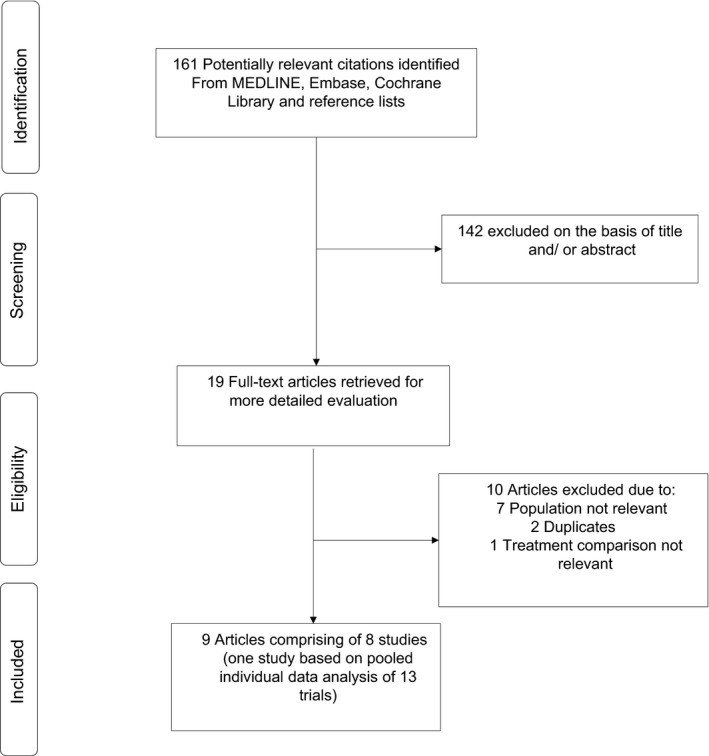
Selection of studies included in the meta‐analysis

Figure 1 shows the study selection process. The search of relevant databases and manual scanning of reference lists of relevant studies identified 161 potentially relevant citations. After the initial screening of titles and abstracts, 19 articles remained for full text evaluation. Following detailed evaluation, 10 articles were excluded because (i) population was not relevant (*n* = 7); (ii) duplicate studies (*n* = 2); and (iii) treatment comparison not relevant (*n* = 1). The remaining nine articles met the inclusion criteria and were included in the review.^28^, ^29^, ^30^, ^31^, ^32^, ^33^, ^34^, ^35^, ^36^

### 3.2 Study characteristics and risk of bias

The nine articles comprised eight studies, of which one was based on a pooled individual patient data (IPD) analysis of 13 trials (Table 1). No RCT specifically investigated the combination of SGLT2 and RAAS inhibitors compared with SGLT2‐Is alone. All eligible studies were based on trials that had investigated the effects of SGLT2 inhibition compared with placebo in patients with type 2 diabetes and reported subgroup results for those treated with or without RAAS‐Is at baseline. Of the 34,551 total participants, 23,109 involved the comparison of SGLT2‐I vs placebo with RAAS inhibition at baseline and 11,442 involved the comparison of SGLT2‐I vs placebo without RAAS inhibition at baseline. In addition to diabetes, patients had other comorbidities such as chronic kidney disease, atherosclerotic CVD or heart failure. Patients had been diagnosed with T2DM and were being managed on standard treatment therapies including ACE‐Is/ARBs, diuretics or calcium channel blockers before inclusion into the trials. All included studies were double‐blinded RCTs. All the studies were conducted in multiple countries. The type of SGLT2‐Is used included dapagliflozin, canagliflozin, empagliflozin, ertugliflozin and sotagliflozin. The majority of trials recruited patients who were at least 18 years old. The average age of participants ranged from 60 to 69 years. The treatment duration ranged from 12 weeks to 6.6 years. Using the Cochrane Collaboration tool, all trials demonstrated low risk of bias in the areas of random sequence generation, allocation concealment, blinding of participants and personnel and incomplete outcome data. Only one trial demonstrated unclear risk of bias for incomplete outcome data and the majority an unclear risk of bias in the areas of selective reporting and other bias (Appendix 3).

**TABLE 1:** Baseline characteristics on eligible studies (2015–2020)

Author, year of publication	Study	Population	Study period	Male %	Average age, years	Age range, years	Country	Intervention	Dose (mg)	Control	Duration	SGLT2‐I / RAAS‐I	SGLT2‐I / Non RAAS‐I
Zinman, 2015; Mayer, 2019	EMPA‐REG OUTCOME	T2DM with prevalent kidney disease	2010–2015	67.8	67.1	≥18	Multinational	Empagliflozin	10 or 25	Placebo	3.1 years	5666	1354
Mancia, 2016	EMPA‐REG BP	T2DM and hypertension	2011–2012	60.1	60.0	≥18	615 sites	Empagliflozin	10 or 25	Placebo	12 weeks	634	190
Neal, 2017	CANVAS Program	T2DM with high cardiovascular risk, eGFR >30	2009, 2014	64.2	63.3	≥30	667 centres in 30 countries	Canagliflozin	100/300	Placebo	188.2 weeks	8116	2026
Cannon, 2020	VERTIS CV	T2DM with atherosclerotic CVD	2013–2019	70.0	64.4	≥40	567 sites in 34 countries	Ertugliflozin	5 or 15	Placebo	3.5 years	6686	1560
Packer, 2020	EMPEROR‐Reduced	HF with or without diabetes	2017–2020	76.1	67.0	≥18	520 sites in 20 countries	Empagliflozin	10	Placebo	16 months	727	3003
Bhatt, 2020a	SCORED	T2DM with CKD and additional cardiovascular risk	2017–2020	55.1	69.0	≥18	750 sites in 44 countries	Sotagliflozin	200/400	Placebo	16 months	118	1990
Bhatt, 2020b	SOLOIST‐WHF	T2DM with worsening HF	2017–2020	66.3	69.0	18–85	306 sites in 32 countries	Sotagliflozin	200/400	Placebo	9 months	205	1017
Scholtes, 2020	Pooled IPD analysis of 13 trials	T2DM with increased albuminuria	2005–2012	63.5	60.1	≥18	Multinational	Dapagliflozin	10	Placebo	12–24 weeks	957	302

Abbreviations: CKD, chronic kidney disease; CVD, cardiovascular disease; HF, heart failure; IPD, individual patient data; SGLT2‐I, sodium‐glucose co‐transporter 2 inhibitor; RAAS‐I, renin‐angiotensin‐aldosterone system inhibitor; T2DM, type 2 diabetes mellitus.

### 3.3 Composite cardiovascular outcome

**FIGURE 2:**
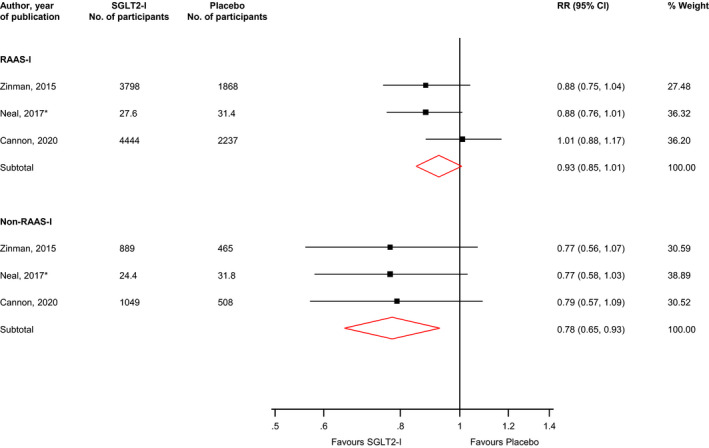
Risk for composite cardiovascular outcome comparing SGLT2 inhibition with placebo in patients with or without RAAS inhibition treatment at baseline. CI, confidence interval (bars); RAAS‐I, renin‐angiotensin‐aldosterone system inhibitor; RR, risk ratio; SGLT2‐I, sodium‐glucose co‐transporter 2 inhibitor; *, number of participants in each treatment arm are reported per 1000 patient years

Comparing SGLT2‐Is with placebo in those on RAAS‐I treatment at baseline, the RR (95% CIs) for the composite cardiovascular outcome in pooled analysis of three trials was 0.93 (0.85–1.01; *I* ^2^ = 13%; 95% CI 0, 91%; *p* for heterogeneity = .32). The corresponding risk in those not on RAAS‐I treatment at baseline was 0.78 (0.65–0.93; *I* ^2^ = 0%; 95% CI 0, 90%; *p* for heterogeneity = .99) (Figure 2). There was no evidence of significant interaction between the effects of SGLT2 inhibition and RAAS inhibition status on the composite cardiovascular outcome (*p*‐value for meta‐regression = .08).

### 3.4 Composite kidney outcome

The composite kidney outcome was reported by only one study. SGLT2‐Is compared with placebo reduced the risk of the composite kidney outcome in those on RAAS inhibition at baseline: 0.52 (95% CI, 0.37–0.74). The corresponding risk for those not treated with RAAS inhibition was 0.65 (95% CI, 0.30–1.39).

### 3.5 Composite outcome of cardiovascular death or HF hospitalization

**FIGURE 3:**
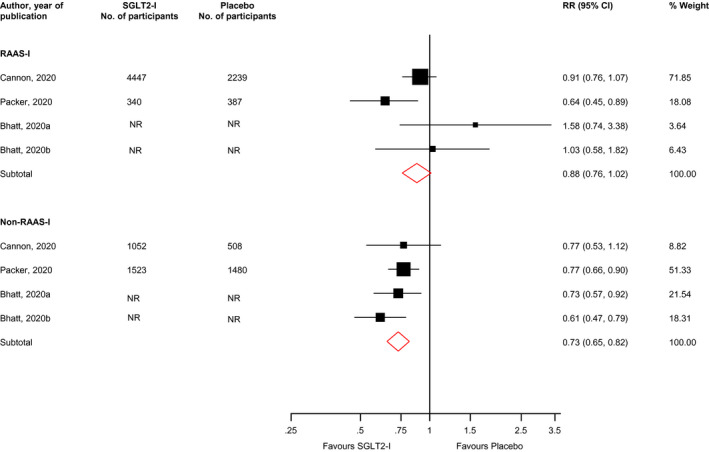
Risk for the composite outcome of cardiovascular death or heart failure hospitalization comparing SGLT2 inhibition with placebo in patients with or without RAAS inhibition treatment at baseline. CI, confidence interval (bars); NR, not reported; RAAS‐I, renin‐angiotensin‐aldosterone system inhibitor; RR, risk ratio; SGLT2‐I, sodium‐glucose co‐transporter 2 inhibitor

In pooled analysis of four trials, the RR (95% CIs) for the composite outcome of cardiovascular death or HF hospitalization was 0.88 (0.76–1.02; *I* ^2^ = 51%; 95% CI 0, 84%; *p* for heterogeneity = .11) when comparing SGLT2‐Is with placebo in those on RAAS‐I treatment at baseline. For those that were not on RAAS inhibition, the corresponding risk was 0.73 (0.65–0.82; *I* ^2^ = 0%; 95% CI 0, 85%; *p* for heterogeneity = .50) (Figure 3). There was no evidence of significant interaction between the effects of SGLT2 inhibition and RAAS inhibition status on the composite outcome of cardiovascular death or HF hospitalization (*p*‐value for meta‐regression = .12).

### 3.6 Cardiovascular death

The outcome of cardiovascular death was reported by one study. SGLT2‐Is compared with placebo reduced the risk of the cardiovascular death in those on RAAS inhibition at baseline: 0.61 (95% CI, 0.48–0.79). The corresponding risk for those not treated with RAAS inhibition was 0.65 (95% CI, 0.39–1.06).

### 3.7 Estimated GFR

Two studies reported the effect of SGLT2 inhibition vs placebo on estimated GFR changes across the subgroup of RAAS‐I users.^29^, ^35^ In the EMPA‐REG OUTCOME trial, the initial change in estimated GFR in patients taking the combination of empagliflozin and RAAS‐Is was higher than for those taking empagliflozin alone (Table 2). The long‐ and post‐treatment changes were also similar for both groups. In the study that pooled data across 13 trials, the effect of dapagliflozin on estimated GFR was similar in patients with or without RAAS inhibition^35^ (Table 2).

**TABLE 2:** Summary of findings of outcomes that could not be pooled

Author, year of publication	Study	Population	Outcome measure	Results	Summary of findings
Mayer, 2019	EMPA‐REG OUTCOME	T2DM with prevalent kidney disease	Change in estimated GFR (baseline to week 4)	Slope per week (95% CI) for RAAS‐I group: −0.879 (−1.001, −0.756) Slope per week (95% CI) for Non RAAS‐I group: −0.368 (−0.618, −0.118).	The change was higher for those taking empagliflozin alone
Mayer, 2019	EMPA‐REG OUTCOME	T2DM with prevalent kidney disease	Change in estimated GFR (week 4 to last value on treatment)	Slope per week (95% CI) for RAAS‐I group: 1.705 (1.330, 2.081) Slope per week (95% CI) for Non RAAS‐I group: 1.630 (0.901, 2.358)	Long‐term treatment changes were similar for both groups
Mayer, 2019	EMPA‐REG OUTCOME	T2DM with prevalent kidney disease	Change in estimated GFR (last value on treatment to follow‐up)	Slope per week (95% CI) for RAAS‐I group: 0.569 (0.441, 0.698) Slope per week (95% CI) for Non RAAS‐I group: 0.586 (0.324, 0.849)	Post treatment changes were similar for both groups
Scholtes, 2020	Pooled IPD analysis of 13 trials	T2DM with increased albuminuria	UACR, estimated GFR, HbA1c and haematocrit	Effect of dapagliflozin on UACR, estimated GFR, HbA1c and haematocrit was similar in patients with or without RAAS inhibition	–
Scholtes, 2020	Pooled IPD analysis of 13 trials	T2DM with increased albuminuria	Body weight, serum uric acid and blood pressure	Mean reductions in body weight, serum uric acid, SBP and DBP were more distinct in patients without RAAS inhibition treatment compared with those with RAAS inhibition treatment at baseline	–
Scholtes, 2020	Pooled IPD analysis of 13 trials	T2DM with increased albuminuria	Adverse effects	Overall, adverse effects were more common in those with RAAS inhibition treatment at baseline compared with those without	–

Abbreviations: DBP, diastolic blood pressure; GFR, glomerular filtration rate; HbA1c, glycated haemoglobin; IPD, individual patient data; SBP, systolic blood pressure; SGLT2‐I, sodium‐glucose co‐transporter 2 inhibitor; RAAS‐I, renin‐angiotensin‐aldosterone system inhibitor; T2DM, type 2 diabetes mellitus; UACR, urinary albumin‐to‐creatinine ratio.

### 3.8 Change in albuminuria

The EMPA‐REG OUTCOME trial reported changes in albuminuria status for the effect of empagliflozin vs placebo across the subgroup of RAAS‐I users.^29^ Though there appeared to be an improvement in albuminuria status in those with baseline RAAS inhibition than those without (Appendix 4), the report noted that there was no significant evidence of interaction across the subgroup. The effect of dapagliflozin on urinary albumin‐to‐creatinine ratio (UACR) was similar in patients with or without RAAS inhibition in the pooled analysis of 13 trials^35^ (Table 2).

### 3.9 Other kidney outcomes

The EMPA‐REG OUTCOME trial reported outcomes for incident or worsening nephropathy, oedema and acute renal failure for the effect of empagliflozin vs placebo across the subgroup of RAAS‐I users.^29^ Empagliflozin compared with placebo reduced the risk of the incident or worsening nephropathy, oedema and acute renal failure in those on RAAS inhibition at baseline; the risk was only reduced for oedema in those who were not on RAAS inhibition treatment (Appendix 5).

### 3.10 Metabolic and haemodynamic parameters

In the pooled analysis of 13 trials, the effect of dapagliflozin on HbA1c and haematocrit was similar in patients with or without RAAS inhibition; however, mean reductions in body weight, serum uric acid, SBP and diastolic blood pressure (DBP) were more distinct in patients without RAAS inhibition treatment compared with those with RAAS inhibition treatment at baseline^35^ (Table 2). The effects of SGLT2 inhibition compared with placebo on the risk of hyperkalaemia, hypokalaemia and hypoglycaemia were similar in both groups (Appendix 6).

### 3.11 Volume depletion

Comparing SGLT2‐Is with placebo in those on RAAS‐I treatment at baseline, the RR (95% CIs) for volume depletion in pooled analysis of two trials was 1.06 (0.84–1.33). The corresponding risk in those not on RAAS‐I treatment at baseline was 0.82 (0.47–1.46) (Appendix 7).

### 3.12 Genital and urinary tract infections

In pooled analysis of two trials, the effect of SGLT2 inhibition vs placebo on genital infections was similar in patients with or without RAAS inhibition (Appendix 8). For urinary tract infection (UTI), the effect of SGLT2 inhibition vs placebo on UTI was also similar in patients with or without RAAS inhibition (Appendix 9).

### 3.13 Other adverse effects

Comparing SGLT2‐Is with placebo in those on RAAS‐I treatment at baseline, the RR (95% CIs) for adverse events in pooled analysis of two trials was 0.99 (0.97–1.00). The corresponding risk in those not on RAAS‐I treatment at baseline was 0.98 (0.95–1.02) (Appendix 10).

### 3.14 GRADE summary of findings

GRADE ratings for the relevant outcomes are reported in a summary of findings table in Appendix 11. GRADE quality of the evidence ranged from very low to moderate.

## 4 DISCUSSION

### 4.1 Key findings

In this systematic review and meta‐analysis from available RCTs, we have evaluated the efficacy and safety outcomes in patients with type 2 diabetes comparing the combination of SGLT2 and RAAS inhibitors with SGLT2‐Is alone. This was achieved by investigating the effects of SGLT2 inhibition compared with placebo in people with type 2 diabetes treated with or without RAAS‐Is at baseline. Our findings show SGLT2 inhibition compared with placebo similarly reduced the risk of major cardiovascular outcomes, improved renal parameters (estimated GFR, volume depletion, changes in albuminuria and electrolyte imbalances) and glycaemic measures (HbA1c and hypoglycaemia) and increased the risk of adverse events including genital infections and UTI in both groups of patients with and without RAAS inhibition. The combination of SGLT2 and RAAS inhibition appeared to reduce the risk of the composite renal outcome, cardiovascular death, incident or worsening nephropathy and acute renal failure, but these results were based on single studies. The study that pooled individual patient data from 13 trials showed distinct reductions in body weight, serum uric acid, SBP and DBP for the combination of SGLT2 and RAAS inhibition than SGLT2 inhibition alone. The quality of the evidence ranged from very low to moderate.

### 4.2 Comparison with previous studies

A previous pooled analysis of individual level data from 13 placebo‐controlled trials investigating the effects of dapagliflozin on cardio‐renal risk factors in patients with type 2 diabetes with increased albuminuria treated with or without RAAS‐Is at baseline reported similar clinically relevant improvements in metabolic and haemodynamic parameters.^35^ In a meta‐analysis of 8 RCTs which compared combined therapy of SGLT2‐Is and ACEIs/ARBs with placebo plus ACEIs/ARBs in patients with type 2 diabetes, the combination therapy showed significant reduction in glycaemic parameters, body weight, blood pressure and lower risk of adverse events.^21^ Another recent meta‐analysis demonstrated that combination therapy with SGLT2‐Is and ACEIs/ARBs compared with ACEIs/ARBs was well‐tolerated and achieved better control of blood pressure, improvement of renal outcomes, alleviation of long‐term renal function and a decrease in blood glucose and body weight, but an increased risk of hypoglycaemia.^22^ To our knowledge, this is the first aggregate meta‐analysis to attempt to evaluate whether the combination of SGLT2 and RAAS inhibitors provides better cardio‐renal clinical outcomes in patients with type 2 diabetes compared with SGLT2‐Is alone. Our overall results suggest that treatment with SGLT2‐Is provides similar clinical effectiveness and safety in patients with type 2 diabetes treated with or without RAAS inhibition. The combination of SGLT2 and RAAS inhibition may improve some renal outcomes and parameters such as body weight and blood pressure compared to SGLT2 inhibition alone, but further evaluation is needed.

### 4.3 Potential explanation of findings

For the past two decades, landmark trials^37^, ^38^ have demonstrated that renin‐angiotensin‐aldosterone system blockade is an efficacious method for the protection of both cardiovascular and renal systems. Despite this, there is some residual risk for both cardiovascular and renal outcomes,^39^ thus necessitating the requirement for further additive treatment options. In our analysis, SGLT2 inhibition compared with placebo reduced the risk of major cardiovascular outcome, but this reduction did not reach statistical significance, as this achievement was expected in a well‐treated population on renin‐angiotensin‐aldosterone system blockade. Thus, in the populations not on RAAS‐Is, the reductions in both composite cardiovascular outcome and composite outcome of cardiovascular death or HF hospitalization were both statistically significant. There is a wealth of clinical data on the renal and cardiovascular protection effects of SGLT2‐Is. They work by targeting target renal tubular glucose reabsorption, thereby exerting glucose lowering effects through glucosuria.^40^ SGLT2‐Is exert renal protection effects in type 2 diabetes by altering renal haemodynamics, reducing intraglomerular pressure, attenuating diabetes‐associated hyperfiltration and tubular hypertrophy, and reducing the tubular toxicity of glucose. They also reduce albuminuria, serum uric acid without potassium abnormalities, blood pressure, afferent arteriole vasoconstriction, osmotic diuresis, weight loss, and the workload of the proximal tubules to improve tubulointerstitial hypoxia, and then allow fibroblasts to resume normal erythropoietin production.^41^ The main functions of the RAAS are regulating fluid volume, blood pressure and the vascular response to injury and inflammation.^42^ Inappropriate activation of the RAAS causes increases in levels of angiotensin II, which lead to end‐organ damage as a result of direct injury to vascular, renal and cardiac tissues. The most commonly used RAAS blockers include ACEIs and ARBs; ACEIs work by reducing the conversion of angiotensin I to angiotensin II, whereas ARBs block the binding of angiotensin II to angiotensin 1 receptor.^43^ These RAAS blockers are effective for treating systemic hypertension, HF and renal insufficiency.^19^ Inhibition of the RAAS constitutes the main therapeutic stay in diabetic nephropathy over the last few decades. These RAAS blockers (ACEIs and ARBs) reduce the incidence of progression to end‐stage kidney disease and major adverse cardiovascular outcomes.^44^

SGLT2 and RAAS inhibitors play different roles at different sites in the kidney, and it has been suggested that their combination might exert synergistic effects on the kidney.^41^ The vasodilatation effect of RAAS‐Is and natriuretic effect of SGLT2‐Is can also complement each other to reduce systemic oxidative stress and inflammation, which can reduce the incidence of cardiovascular events.^45^ Several clinical studies have indicated that the combination of SGLT2‐Is with ACEIs/ARBs was associated with greater cardio‐ and reno‐protection and improvement in glycaemic measures, blood pressure and body weight and was well tolerated.^21^, ^22^, ^46^, ^47^ It may appear that our findings are at odds with the existing evidence, but this is likely because our evaluation which was mainly based on study level subgroup analyses, precluded a head‐to‐head comparison between the combination therapy (SGLT2 plus RAAS inhibitors) and SGLT2‐I. Furthermore, our analysis was limited by the few studies available for pooling. Nevertheless, our findings do suggest that the combination of SGLT2 and RAAS inhibitors may be similar in efficacy and safety if not superior to SGLT2‐Is alone.

### 4.4 Implications of findings

Our overall study findings show that the combination of SGLT2 and RAAS inhibitors may have similar cardiovascular and renal benefits in patients with type 2 diabetes compared with SGLT2 inhibitors alone. There is a likelihood that the combination of SGLT2 and RAAS inhibitors may be superior compared to SGLT2‐Is alone in the prevention of renal deterioration in addition to improving body weight and blood pressure, though further data are needed to confirm this. With the rapid increase in the prevalence of type 2 diabetes globally because of increasingly poor lifestyle choices, morbidity and deaths attributable to diabetes will experience a steep increase. A large armamentarium of therapeutic options is urgently needed for the management of type 2 diabetes. For the last two decades, pharmacological inhibition of the RAAS using RAAS‐Is has been the major focus for the management of diabetes nephropathy, which has been associated with good results. The RAAS‐Is have also been used for their cardioprotective effects. Previous studies have shown that combined therapy of SGLT2 and RAAS inhibitors is superior to RAAS‐I therapy alone in patients with type 2 diabetes.^21^, ^22^ Taken, the overall results together suggest that SGLT2 inhibition has superior cardio and reno‐protective effects over RAAS inhibition in type 2 diabetes treatment. The use of SGLT2 inhibition as a first line therapy in type 2 diabetes or its early use in the prevention of renal deterioration and cardiovascular complications in addition to its glycaemic control deserves further study. The absence of a significant benefit of the combination of SGLT2‐I and RAAS‐Is on both composite cardiovascular outcome and composite outcome of cardiovascular death or HF hospitalization leaves room for the use of only SGLT2‐Is in populations that may be struggling with polypharmacy and de‐prescribing of some agents necessary. In these situations, the use of only SGLT2‐Is could yield similar outcomes as the combination.

### 4.5 Strengths and limitations

The strengths of the current evaluation deserve consideration. First is the novelty; though a number of RCTs comparing SGLT2‐Is with placebo have reported outcomes among subgroups of patients with or without RAAS inhibition, no review has previously synthesized the evidence. Previous reviews have rather compared combined therapy of SGLT2‐Is and ACEIs/ARBs with ACEIs/ARBs in patients with type 2 diabetes.^21^, ^22^ Second, our population of study was clearly defined, which was based on patients with type 2 diabetes treated with or without RAAS inhibition at baseline. Third, our review was prespecified to include only RCTs, which represent the gold standard study designs for evaluating the effectiveness of interventions. Fourth, to minimize selective reporting, we evaluated a comprehensive panel of efficacy and safety outcomes as reported by the individual studies. Finally, we conducted interaction analyses where possible to assess statistical differences in the effect of the two interventions (SGLT2 plus RAAS inhibition vs SGLT2 inhibition). The limitations were inherent and unavoidable. Though we performed quantitative synthesis of the data where possible, inconsistent reporting of outcome measures from some of the studies and findings based on single reports precluded pooling of all available data. Most of the data were based on subgroup analyses reported by the trials, which may be misleading. A head‐to‐head comparison of the two interventions was not possible. Definitive trials that are powered to compare the combination of SGLT2 and RAAS inhibition with SGLT2‐Is alone in people with type 2 diabetes are warranted.

## 5 CONCLUSIONS

In conclusion, emerging data suggest that the combination of SGLT2 and RAAS inhibitors appear to have similar cardiovascular and renal benefits in patients with type 2 diabetes compared with SGLT2 inhibitors alone. The combination of SGLT2 and RAAS inhibition may have superior benefits which include reductions in body weight and blood pressure and reducing the risk of renal outcomes such as nephropathy and acute renal failure, but further data based on head‐to‐head comparisons are needed.

## CONFLICT OF INTEREST

Dr. Khunti reports personal fees from Amgen, personal fees from Astrazeneca, personal fees from Bayer, personal fees from NAPP, personal fees from Lilly, personal fees from Merck Sharp & Dohme, personal fees from Novartis, personal fees from Novo Nordisk, personal fees from Roche, personal fees from Berlin‐Chemie AG / Menarini Group, personal fees from Sanofi‐Aventis, personal fees from Servier, personal fees from Boehringer Ingelheim, grants from Pfizer, grants from Boehringer Ingelheim, grants from AstraZeneca, grants from Novartis, grants from Novo Nordisk, grants from Sanofi‐Aventis, grants from Lilly, grants from Merck Sharp & Dohme, grants from Servier, outside the submitted work. Dr. Seidu reports personal fees from Amgen, personal fees from Astrazeneca, personal fees from NAPP, personal fees from Lilly, personal fees from Merck Sharp & Dohme, personal fees from Novartis, personal fees from Novo Nordisk, personal fees from Roche, personal fees from Sanofi‐Aventis, personal fees from Boehringer Ingelheim, grants from AstraZeneca, grants from Sanofi‐Aventis, grants from Servier, grants from Janssen, outside the submitted work. Dr Kunutor has no conflicts to report. Dr Topsever has received educational sponsorship or honoraria for speaking at meetings or serving at Advisory Boards from Boehringer Ingelheim, Lilly, Novo Nordisk and Sanofi Aventis.

## AUTHOR CONTRIBUTIONS

**Samuel Seidu:** Conceptualization (lead); Data curation (equal); Formal analysis (equal); Funding acquisition (lead); Investigation (lead); Methodology (lead); Project administration (lead); Resources (lead); Software (equal); Supervision (equal); Validation (equal); Visualization (equal); Writing‐original draft (equal); Writing‐review & editing (equal). **Setor Kunutsor:** Conceptualization (supporting); Data curation (equal); Formal analysis (equal); Funding acquisition (supporting); Investigation (equal); Methodology (equal); Project administration (supporting); Resources (supporting); Software (equal); Supervision (supporting); Validation (equal); Visualization (equal); Writing‐original draft (lead); Writing‐review & editing (lead). **PINAR TOPSEVER:** Conceptualization (supporting); Data curation (supporting); Formal analysis (supporting); Funding acquisition (supporting); Investigation (supporting); Methodology (supporting); Project administration (supporting); Resources (supporting); Software (supporting); Supervision (supporting); Validation (equal); Visualization (equal); Writing‐original draft (supporting); Writing‐review & editing (equal). **Kamlesh Khunti:** Conceptualization (equal); Data curation (supporting); Formal analysis (equal); Funding acquisition (supporting); Investigation (supporting); Methodology (equal); Project administration (equal); Resources (equal); Software (supporting); Supervision (lead); Validation (equal); Visualization (supporting); Writing‐original draft (supporting); Writing‐review & editing (equal).

## Supporting information

Appendix S1‐S11

## Data Availability

The corresponding author had full access to all the data in the study and takes responsibility for the integrity of the data and the accuracy of the data analysis. This study is based in data from published articles.
