# Supplementary material for: Benefits and harms of sodium‐glucose co‐transporter‐2 inhibitors (SGLT2‐I) and renin–angiotensin–aldosterone system inhibitors (RAAS‐I) versus SGLT2‐Is alone in patients with type 2 diabetes: A systematic review and meta‐analysis of randomized controlled trials
Source: Endocrinol Diabetes Metab. 2021 Oct 12;5(1):e00303. doi: 10.1002/edm2.303 (PMC8754244; doi:10.1002/edm2.303)
Supplement: Supplementary file 1 — Appendix S1‐S11 [file EDM2-5-e00303-s001.docx]

**SUPPLEMENTARY MATERIAL**

| **Appendix 1** | PRISMA checklist |
| --- | --- |
| **Appendix 2** | MEDLINE literature search strategy |
| **Appendix 3** | Assessment of risk of bias |
| **Appendix 4** | Risk for change in albuminuria comparing SGLT2 inhibition with placebo in patients with or without RAAS inhibition treatment at baseline |
| **Appendix 5** | Risk for other renal outcomes comparing SGLT2 inhibition with placebo in patients with or without RAAS inhibition treatment at baseline |
| **Appendix 6** | Risk for metabolic parameters comparing SGLT2 inhibition with placebo in patients with or without RAAS inhibition treatment at baseline |
| **Appendix 7** | Risk for volume depletion comparing SGLT2 inhibition with placebo in patients with or without RAAS inhibition treatment at baseline |
| **Appendix 8** | Risk for genital infections comparing SGLT2 inhibition with placebo in patients with or without RAAS inhibition treatment at baseline |
| **Appendix 9** | Risk for urinary tract infection comparing SGLT2 inhibition with placebo in patients with or without RAAS inhibition treatment at baseline |
| **Appendix 10** | Risk for adverse events comparing SGLT2 inhibition with placebo in patients with or without RAAS inhibition treatment at baseline |
| **Appendix 11** | GRADE summary of findings |

**Appendix 1:** PRISMA checklist

| **Section/topic** | **Item No** | **Checklist item** | **Reported on page No** |
| --- | --- | --- | --- |
| **Title** | | | |
| Title | 1 | Identify the report as a systematic review, meta-analysis, or both | 1 |
| **Abstract** | | | |
| Structured summary | 2 | Provide a structured summary including, as applicable, background, objectives, data sources, study eligibility criteria, participants, interventions, study appraisal and synthesis methods, results, limitations, conclusions and implications of key findings, systematic review registration number | 2 |
| **Introduction** | | | |
| Rationale | 3 | Describe the rationale for the review in the context of what is already known | Introduction |
| Objectives | 4 | Provide an explicit statement of questions being addressed with reference to participants, interventions, comparisons, outcomes, and study design (PICOS) | Introduction |
| **Methods** | | | |
| Protocol and registration | 5 | Indicate if a review protocol exists, if and where it can be accessed (such as web address), and, if available, provide registration information including registration number | Methods |
| Eligibility criteria | 6 | Specify study characteristics (such as PICOS, length of follow-up) and report characteristics (such as years considered, language, publication status) used as criteria for eligibility, giving rationale | Methods |
| Information sources | 7 | Describe all information sources (such as databases with dates of coverage, contact with study authors to identify additional studies) in the search and date last searched | Methods |
| Search | 8 | Present full electronic search strategy for at least one database, including any limits used, such that it could be repeated | Appendix 2 |
| Study selection | 9 | State the process for selecting studies (that is, screening, eligibility, included in systematic review, and, if applicable, included in the meta-analysis) | Methods |
| Data collection process | 10 | Describe method of data extraction from reports (such as piloted forms, independently, in duplicate) and any processes for obtaining and confirming data from investigators | Methods |
| Data items | 11 | List and define all variables for which data were sought (such as PICOS, funding sources) and any assumptions and simplifications made | Methods |
| Risk of bias in individual studies | 12 | Describe methods used for assessing risk of bias of individual studies (including specification of whether this was done at the study or outcome level), and how this information is to be used in any data synthesis | Methods |
| Summary measures | 13 | State the principal summary measures (such as risk ratio, difference in means). | Methods |
| Synthesis of results | 14 | Describe the methods of handling data and combining results of studies, if done, including measures of consistency (such as I^2^ statistic) for each meta-analysis | Methods |
| Risk of bias across studies | 15 | Specify any assessment of risk of bias that may affect the cumulative evidence (such as publication bias, selective reporting within studies) | Methods |
| Additional analyses | 16 | Describe methods of additional analyses (such as sensitivity or subgroup analyses, meta-regression), if done, indicating which were pre-specified | Methods |
| **Results** | | | |
| Study selection | 17 | Give numbers of studies screened, assessed for eligibility, and included in the review, with reasons for exclusions at each stage, ideally with a flow diagram | Results, Figure 1 |
| Study characteristics | 18 | For each study, present characteristics for which data were extracted (such as study size, PICOS, follow-up period) and provide the citations | Results, Table 1 |
| Risk of bias within studies | 19 | Present data on risk of bias of each study and, if available, any outcome-level assessment (see item 12). | Results, Appendix 3 |
| Results of individual studies | 20 | For all outcomes considered (benefits or harms), present for each study (a) simple summary data for each intervention group and (b) effect estimates and confidence intervals, ideally with a forest plot |  |
| Synthesis of results | 21 | Present results of each meta-analysis done, including confidence intervals and measures of consistency | Results, Figures 2-4; Appendices 4-10 |
| Risk of bias across studies | 22 | Present results of any assessment of risk of bias across studies (see item 15) | Not applicable |
| Additional analysis | 23 | Give results of additional analyses, if done (such as sensitivity or subgroup analyses, meta-regression) (see item 16) | Results |
| **Discussion** | | | |
| Summary of evidence | 24 | Summarise the main findings including the strength of evidence for each main outcome; consider their relevance to key groups (such as health care providers, users, and policy makers) | Discussion |
| Limitations | 25 | Discuss limitations at study and outcome level (such as risk of bias), and at review level (such as incomplete retrieval of identified research, reporting bias) | Discussion |
| Conclusions | 26 | Provide a general interpretation of the results in the context of other evidence, and implications for future research | Discussion |
| **Funding** | | | |
| Funding | 27 | Describe sources of funding for the systematic review and other support (such as supply of data) and role of funders for the systematic review | After Discussion |

**Appendix 2:** MEDLINE literature search strategy

1 dapagliflozin.mp. (1410)

2 exp Canagliflozin/ad, ae, tu [Administration & Dosage, Adverse Effects, Therapeutic Use] (430)

3 empagliflozin.mp. (1584)

4 ertugliflozin.mp. (129)

5 sotagliflozin.mp. (86)

6 SGLT2 inhibitor.mp. or exp Sodium-Glucose Transporter 2 Inhibitors/ (3735)

7 exp Diabetes Mellitus, Type 2/ (140335)

8 diabetes.mp. or exp Diabetes Mellitus/ (680379)

9 ACEI*.mp. (5450)

10 ARB*.mp. (106534)

11 renin angiotensin system inhibitor*.mp. (710)

12 renin angiotensin aldosterone system inhibitor*.mp. (409)

13 exp Angiotensin II Type 1 Receptor Blockers/ (18199)

14 ACE inhibitor*.mp. (17907)

15 RAS inhibitor*.mp. (915)

16 RAAS inhibitor*.mp. (350)

17 exp Angiotensin-Converting Enzyme Inhibitors/ (44705)

18 renin angiotensin system inhibitor*.mp. (710)

19 exp Angiotensin Receptor Antagonists/ or angiotensin receptor blocker*.mp. (28319)

20 direct renin inhibitor*.mp. (534)

21 ("clinical trial" or "clinical trial, phase i" or "clinical trial, phase ii" or clinical trial, phase iii or clinical trial, phase iv or controlled clinical trial or "multicenter study" or "randomized controlled trial").pt. or double-blind method/ or clinical trials as topic/ or clinical trials, phase i as topic/ or clinical trials, phase ii as topic/ or clinical trials, phase iii as topic/ or clinical trials, phase iv as topic/ or controlled clinical trials as topic/ or randomized controlled trials as topic/ or early termination of clinical trials as topic/ or multicenter studies as topic/ or ((randomi?ed adj7 trial*) or (controlled adj3 trial*) or (clinical adj2 trial*) or ((single or doubl* or tripl* or treb*) and (blind* or mask*))).ti,ab,kw. or ("4 arm" or "four arm").ti,ab,kw. (1706918)

22 1 or 2 or 3 or 4 or 5 or 6 (4961)

23 7 or 8 (680379)

24 9 or 10 or 11 or 12 or 13 or 14 or 15 or 16 or 17 or 18 or 19 or 20 (172134)

25 21 and 22 and 23 and 24 (32)

26 limit 25 to yr="2012 -Current" (32)

Each part was specifically translated for searching alternative databases.

**Appendix 3:** Assessment of risk of bias

**Appendix 4:** Risk for change in albuminuria comparing SGLT2 inhibition with placebo in patients with or without RAAS inhibition treatment at baseline

CI, confidence interval (bars); RAAS, renin-angiotensin-aldosterone system; RR, risk ratio; SGLT2, sodium–glucose co-transporter 2

**Appendix 5:** Risk for other renal outcomes comparing SGLT2 inhibition with placebo in patients with or without RAAS inhibition treatment at baseline

CI, confidence interval (bars); RAAS, renin-angiotensin-aldosterone system; RR, risk ratio; SGLT2, sodium–glucose co-transporter 2

**Appendix 6:** Risk for metabolic parameters comparing SGLT2 inhibition with placebo in patients with or without RAAS inhibition treatment at baseline

CI, confidence interval (bars); RAAS, renin-angiotensin-aldosterone system; RR, risk ratio; SGLT2, sodium–glucose co-transporter 2

**Appendix 7:** Risk for volume depletion comparing SGLT2 inhibition with placebo in patients with or without RAAS inhibition treatment at baseline

CI, confidence interval (bars); RAAS, renin-angiotensin-aldosterone system; RR, risk ratio; SGLT2, sodium–glucose co-transporter 2

**Appendix 8:** Risk for genital infections comparing SGLT2 inhibition with placebo in patients with or without RAAS inhibition treatment at baseline

CI, confidence interval (bars); RAAS, renin-angiotensin-aldosterone system; RR, risk ratio; SGLT2, sodium–glucose co-transporter 2

**Appendix 9:** Risk for urinary tract infections comparing SGLT2 inhibition with placebo in patients with or without RAAS inhibition treatment at baseline

CI, confidence interval (bars); RAAS, renin-angiotensin-aldosterone system; RR, risk ratio; SGLT2, sodium–glucose co-transporter 2

**Appendix 10:** Risk for other adverse events comparing SGLT2 inhibition with placebo in patients with or without RAAS inhibition treatment at baseline

CI, confidence interval (bars); RAAS, renin-angiotensin-aldosterone system; RR, risk ratio; SGLT2, sodium–glucose co-transporter 2

**Appendix 11:** GRADE summary of findings

| **Certainty assessment** | | | | | | | **№ of patients** | | **Effect** | | **Certainty** | **Importance** |
| --- | --- | --- | --- | --- | --- | --- | --- | --- | --- | --- | --- | --- |
| **№ of studies** | **Study design** | **Risk of bias** | **Inconsistency** | **Indirectness** | **Imprecision** | **Other considerations** | **[SGLT2 inhibitor]** | **[Placebo]** | **Relative (95% CI)** | **Absolute (95% CI)** |  |  |
| **Composite cardiovascular outcome for those on RAAS inhibitors** | | | | | | | | | | | | |
| 3 | randomised trials | not serious | not serious | serious ^a^ | not serious | none |  |  | **RR 0.93** (0.85 to 1.01) | **1 fewer per 1,000** (from 1 fewer to 1 fewer) | ⨁⨁⨁◯ MODERATE |  |
| **Composite cardiovascular outcome for those not on RAAS inhibitors** | | | | | | | | | | | | |
| 3 | randomised trials | not serious | not serious | serious ^a^ | not serious | none |  |  | **RR 0.78** (0.65 to 0.93) | **1 fewer per 1,000** (from 1 fewer to 1 fewer) | ⨁⨁⨁◯ MODERATE |  |
| **Composite outcome of CVD death/HF hospitalization for those on RAAS inhibitors** | | | | | | | | | | | | |
| 4 | randomised trials | not serious | serious ^b^ | serious ^a^ | not serious | none |  |  | **RR 0.88** (0.76 to 1.02) | **1 fewer per 1,000** (from 1 fewer to 1 fewer) | ⨁⨁◯◯ LOW |  |
| **Composite outcome of CVD death/HF hospitalization for those not on RAAS inhibitors** | | | | | | | | | | | | |
| 4 | randomised trials | not serious | not serious | serious ^a^ | not serious | none |  |  | **RR 0.73** (0.65 to 0.82) | **1 fewer per 1,000** (from 1 fewer to 1 fewer) | ⨁⨁⨁◯ MODERATE |  |
| **Volume depletion for those on RAAS inhibitors** | | | | | | | | | | | | |
| 2 | randomised trials | not serious | not serious | serious ^a^ | serious ^c^ | none |  |  | **RR 1.06** (0.84 to 1.33) | **1 fewer per 1,000** (from 1 fewer to 1 fewer) | ⨁⨁◯◯ LOW |  |
| **Volume depletion for those not on RAAS inhibitors** | | | | | | | | | | | | |
| 2 | randomised trials | not serious | not serious | serious ^a^ | serious ^c^ | none |  |  | **RR 0.82** (0.47 to 1.46) | **1 fewer per 1,000** (from 1 fewer to 0 fewer) | ⨁⨁◯◯ LOW |  |
| **Genital infections for those on RAAS inhibitors** | | | | | | | | | | | | |
| 2 | randomised trials | not serious | not serious | serious ^a^ | very serious ^d^ | none |  |  | **RR 3.66** (2.58 to 5.18) | **4 fewer per 1,000** (from 5 fewer to 3 fewer) | ⨁◯◯◯ VERY LOW |  |
| **Genital infections for those not on RAAS inhibitors** | | | | | | | | | | | | |
| 2 | randomised trials | not serious | not serious | serious ^a^ | very serious ^d^ | none |  |  | **RR 3.85** (1.89 to 7.82) | **4 fewer per 1,000** (from 8 fewer to 2 fewer) | ⨁◯◯◯ VERY LOW |  |
| **Urinary tract infections for those on RAAS inhibitors** | | | | | | | | | | | | |
| 2 | randomised trials | not serious | not serious | serious ^a^ | not serious | none |  |  | **RR 0.99** (0.88 to 1.11) | **1 fewer per 1,000** (from 1 fewer to 1 fewer) | ⨁⨁⨁◯ MODERATE |  |
| **Urinary tract infections for those not on RAAS inhibitors** | | | | | | | | | | | | |
| 2 | randomised trials | not serious | not serious | serious ^a^ | not serious | none |  |  | **RR 1.04** (0.82 to 1.31) | **1 fewer per 1,000** (from 1 fewer to 1 fewer) | ⨁⨁⨁◯ MODERATE |  |
| **Adverse effects for those on RAAS inhibitors** | | | | | | | | | | | | |
| 2 | randomised trials | not serious | not serious | serious ^a^ | not serious | none |  |  | **RR 0.99** (0.97 to 1.00) | **1 fewer per 1,000** (from 1 fewer to 1 fewer) | ⨁⨁⨁◯ MODERATE |  |
| **Adverse effects for those not on RAAS inhibitors** | | | | | | | | | | | | |
| 2 | randomised trials | not serious | not serious | serious ^a^ | not serious | none |  |  | **RR 0.98** (0.95 to 1.02) | **1 fewer per 1,000** (from 1 fewer to 1 fewer) | ⨁⨁⨁◯ MODERATE |  |

CI, confidence interval; CVD, cardiovascular disease; RAAS, renin-angiotensin-aldosterone system; RR, risk ratio; SGLT2, sodium–glucose co-transporter 2

#### Explanations

a. Results based on subgroup analyses

b. I-squared value of 51%

c. Wide confidence interval

d. Very wide confidence interval
